# Supplementary material for: Applying GRADE-CERQual to qualitative evidence synthesis findings—paper 4: how to assess coherence
Source: Implement Sci. 2018 Jan 25;13(Suppl 1):13. doi: 10.1186/s13012-017-0691-8 (PMC5791039; doi:10.1186/s13012-017-0691-8)
Supplement: Supplementary file 1 — Key definitions relevant to CERQual. (PDF 619 kb) [file 13012_2017_691_MOESM1_ESM.pdf]

Additional file 1: Key definitions relevant to CERQual

Adequacy of data: an overall determination of the degree of richness as well as the quantity of data supporting a review finding [1].

Coherence of the review finding: how clear and cogent the fit is between the data from the primary studies and a review finding that synthesizes that data [2].

Confidence in the evidence: an assessment of the extent to which a review finding is a reasonable representation of the phenomenon of interest [1].

Dissemination bias in qualitative research: a systematic distortion of the phenomenon of interest due to selective dissemination of qualitative studies or the findings of qualitative studies [1].

Methodological limitations: the extent to which there are concerns about the design or conduct of the primary studies that contributed evidence to an individual review finding [1].

Phenomenon of interest: the issue that is the focus of the qualitative inquiry – that is, "what we want our research to understand…[]… explain, or describe'' [3](p129).

Relevance: the extent to which the body of data from the primary studies supporting a review finding is applicable to the context specified in the review question [1].

Review finding: An analytic output (e.g., a theme, category, thematic framework, theory or contribution to theory) from a qualitative evidence synthesis that, based on data from primary studies, describes a phenomenon or an aspect of a phenomenon [1].

References

1. Lewin S, Glenton C, Munthe-Kaas H, Carlsen B, Colvin CJ, Gulmezoglu M, Noyes J, Booth A, Garside R, Rashidian A: **Using qualitative evidence in decision making for health and social interventions: an approach to assess confidence in findings from qualitative evidence syntheses (GRADE-CERQual)**. *PLoS Med* 2015, **12**(10):e1001895.
2. Colvin CJ, Garside R, Wainwright M, Lewin S, Bohren M, Glenton C, Munthe-Kaas HM, Carlsen B, Tuncalp Ö, Noyes J *et al*: **Applying GRADE-CERQual to qualitative evidence synthesis findings - paper 4 of 7: how to assess coherence**. *Implementation Science* 2017, **12 Suppl 2**.
3. Rappaport J: **Terms of empowerment/exemplars of prevention: toward a theory for community psychology**. *Am J Community Psychol* 1987, **15**(2):121-148.
